# Effectiveness of PIVKA-II in the detection of hepatocellular carcinoma based on real-world clinical data

**DOI:** 10.1186/s12885-017-3609-6

**Published:** 2017-09-01

**Authors:** Rentao Yu, Zhaoxia Tan, Xiaomei Xiang, Yunjie Dan, Guohong Deng

**Affiliations:** 1 Department of Infectious Diseases, Southwest Hospital, Third Military Medical University, Chongqing, 400038 China; 2 Chongqing Key Laboratory of Infectious Diseases, Southwest Hospital, Third Military Medical University, Chongqing, 400038 China; 3 Institute of Immunology, Third Military Medical University, Chongqing, 400038 China

**Keywords:** PIVKA-II, HCC, Real-world, AFP, Surveillance

## Abstract

**Background:**

Protein Induced by Vitamin K Absence or Antagonist-II (PIVKA-II) is an efficient biomarker specific for hepatocellular carcinoma (HCC). Some researchers have proved that levels of PIVKA-II reflect HCC oncogenesis and progression. However, the effectiveness of PIVKA-II based on real-world clnical data has barely been studied.

**Methods:**

A total of 14,861 samples were tested in Southwest Hospital in over 2 years’ time. Among them, 4073 samples were PIVKA-II positive. Finally, a total of 2070 patients with at least two image examinations were enrolled in this study. Levels of AFP and PIVKA-II were measured by chemiluminescence enzyme immunoassay (CLEIA) and chemiluminescent microparticle Immunoassay (CMIA), respectively.

**Results:**

A total of 1016 patients with HCC were detected by PIVKA-II in a real-world application. In all these cases, 88.7% cases primarily occurred and patients with advanced HCC covered 61.3%. Levels of PIVKA-II were significantly higher in advanced group (4650.0 mAU/ml, 667.0–33,438.0 mAU/ml) than early-stage group (104.5 mAU/ml, 61.0–348.8 mAU/ml; *P* < 0.001). Levels of PIVKA-II elevated significantly in recurrence and residual group than recovery group (*P* < 0.001). A total of 1054 PIVKA-II positive patients were non-HCC cases. Among them, cirrhosis took the largest part (46.3%), followed by hepatitis (20.6%) and benign nodules (15.3%). High-levels of PIVKA-II in at-risk patients is an indicator of HCC development in two-year time.

**Conclusions:**

Our data showed that PIVKA-II effectively increases the detection rate of HCC was a valid complement to AFP and image examination in HCC surveillance.

## Background

Recent years have witnessed a huge decrease in cancer mortality rate due to the progression of cancer treatment [1–3], especially with the development of next-generation sequencing, immune therapy and targeted drugs [4–6]. However, things are different in the area of hepatocellular carcinoma (HCC). Due to the inadequate approaches of early detection, around 50% of HCC cases were diagnosed at late stage when the 5-year overall survival rate is lower than 10% [7]. Chronic hepatitis B virus (HBV) infection ranks the major cause of HCC in Asia and sub-Saharan Africa [8, 9]. Researchers have proven that antiviral treatment reduces the risk of HCC [10–12]. However, eliminating the risk of HCC in chronic hepatitis B (CHB) patients has a long way to go. Under this circumstance, there is a strong need for a feasible surveillance strategy for at-risk populations to increase the early detection rate of HCC.

Protein Induced by Vitamin K Absence or Antagonist-II (PIVKA-II), also known as Des-γ -carboxy-prothrombin (DCP), is believed to be a suitable serum biomarker specific for HCC since first detected by Libert et al. at 1984 [13]. With the development of accurate measuring methods [14, 15], PIVKA-II has been recommended as one of a surveillance method for HCC in at-risk populations and written into the guidelines of the Japan Society of Hepatology (JSH) [16, 17].

Clinical researches have revealed that alpha-fetoprotein(AFP) combined with PIVKA-II elevated the detection rate of about 8–20% with a satisfactory sensitivity and specificity [18–21]. As for HCC prognosis, treatment response and recurrence monitoring, PIVKA-II could also improve the performance [22–24]. However, all these studies were designed in reasonable ways with cases and controls limited to certain groups of people. In real-world settings, different people with different conditions and backgrounds may have great influence on the levels of PIVKA-II. However, the effectiveness of PIVKA-II in detecting HCC based on real-world clinical data has barely been studied.

## Methods

### Study populations

**Fig. 1:**
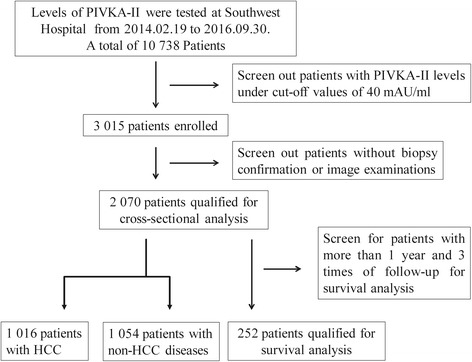
Flow diagram of the selection procedure. A cross-sectional study was conducted in PIVKA-II (+) patients with pathological or imaging confirmation. Survival analysis was conducted based on confirmed populations with follow-up

Figure 1 shows the selection flow of this study. Between Feb 2014 and Sep 2016, 10,738 at-risk patients (a total of 14,861 samples) visiting Southwest Hospital were tested the levels of PIVKA-II. Among them, 4073 samples (3015 patients) were PIVKA-II positive (cut-off: 40 mAU/ml). Finally, a total of 2070 patients with at least two image examinations or biopsy were enrolled in this study for cross-sectional analysis, of which 1016 patients (covered 49.1% of all PIVKA-II+ patients) were HCC cases and another 1054 PIVKA-II positive patients were non-HCC cases. For survival analysis, patients with more than 1 years and 3 times of follow-ups were recruited and 252 patients met the criterion and were enrolled.

The diagnosis of each case was ascertained by image tests and a few of them were undertaken further pathological examinations. The diagnosis of HCC was determined by at least two enhanced image examinations, enhanced computed tomography (CT)/enhanced magnetic resonance imaging (MRI)/ultrasonography (USG), or by pathological confirmation. For cross-sectional analysis, PIVKA-II levels in HCC group were selected at the time of image diagnosis, while in the non-HCC group, PIVKA-II levels of the last result were selected for analysis. For survival analysis, PIVKA-II levels at baseline point and all follow-up points were analyzed. All clinical data were grabbed from electronic medical record system of Southwest Hospital.

### Measurement of PIVKA-II and AFP

Serum levels of PIVKA-II were determined by chemiluminescence enzyme immunoassay (CLEIA) (LUMIPULSE® G1200, FUJIREBIO INC., Japan). The cut-off value was 40 mAU/ml. Serum levels of AFP were measured by AFP Reagent kit via chemiluminescent microparticle immunoassay (CMIA) ARCHITECT i2000, Abbott Laboratories, America). The cut-off value was set at 20 ng/ml.

### Statistical analysis

SPSS version 22.0 statistical software (IBM, USA) and MedCalc version 11.4.2.0 (MedCalc Software bvba, Belgium) were applied for all statistical analysis and the graphs were constructed on the Prism version 6.00 (GraphPad Software Inc., USA). Each variable was represented as median with interquartile range. For cross-sectional analysis, normality and homogeneity of all data were evaluated by Kolmogorov–Smirnov test. Student T-test or Mann-Whitney test was applied to compare the differences between two categorical variables and for multi-categorical variables, one-way ANOVA or Kruskal-Wallis test was used. Sensitivity, specificity, Kappa value and diagnostic accuracy were calculated by 2 × 2 table in SPSS. Pearson Chi-square test was employed to evaluate statistical differences of diagnostic performance at different cut-off values. Receiver Operating Characteristics (ROC) Curves and area under ROC (AUROC) were calculated to evaluate the detecting efficiency of PIVKA-II, and DeLong test was applied to compare the different AUROC. For survival analysis, the cumulative incidence of HCC by patient groups with different levels of PIVKA-II was assessed with Kaplan-Meier analyses, and crude differences were calculated by log-rank test. Cox proportional hazard models were used to calculate hazard ratios and 95% confidence intervals of HCC. Covariates with a *P* value less than 0.1 in univariate analysis were included in multivariate analysis. Two-tailed *P* value less than 0.05 was defined to be statistically significant.

## Results

### Effectiveness of PIVKA-II in diagnosing HCC

In about two and a half years’ time, a total of 1016 patients with HCC (covered 49.1% of all PIVKA-II+ patients) were detected by PIVKA-II in the clinical application at Southwest Hospital, Chongqing, China. Among these diagnosed HCC patients, serum AFP (cut-off: 20 ng/ml) levels in 230 cases (22.6%) were negative at the time of diagnosis. Besides, 241 cancer cases (23.7%) of PIVKA-II positive presented no signs of tumor in image examination the first time but were diagnosed as HCC later. The average gap between the elevation of PIVKA-II level and positive results in image examination was 402.5 ± 192.3 days.

### Distribution of all cases of different diseases

**Fig. 2:**
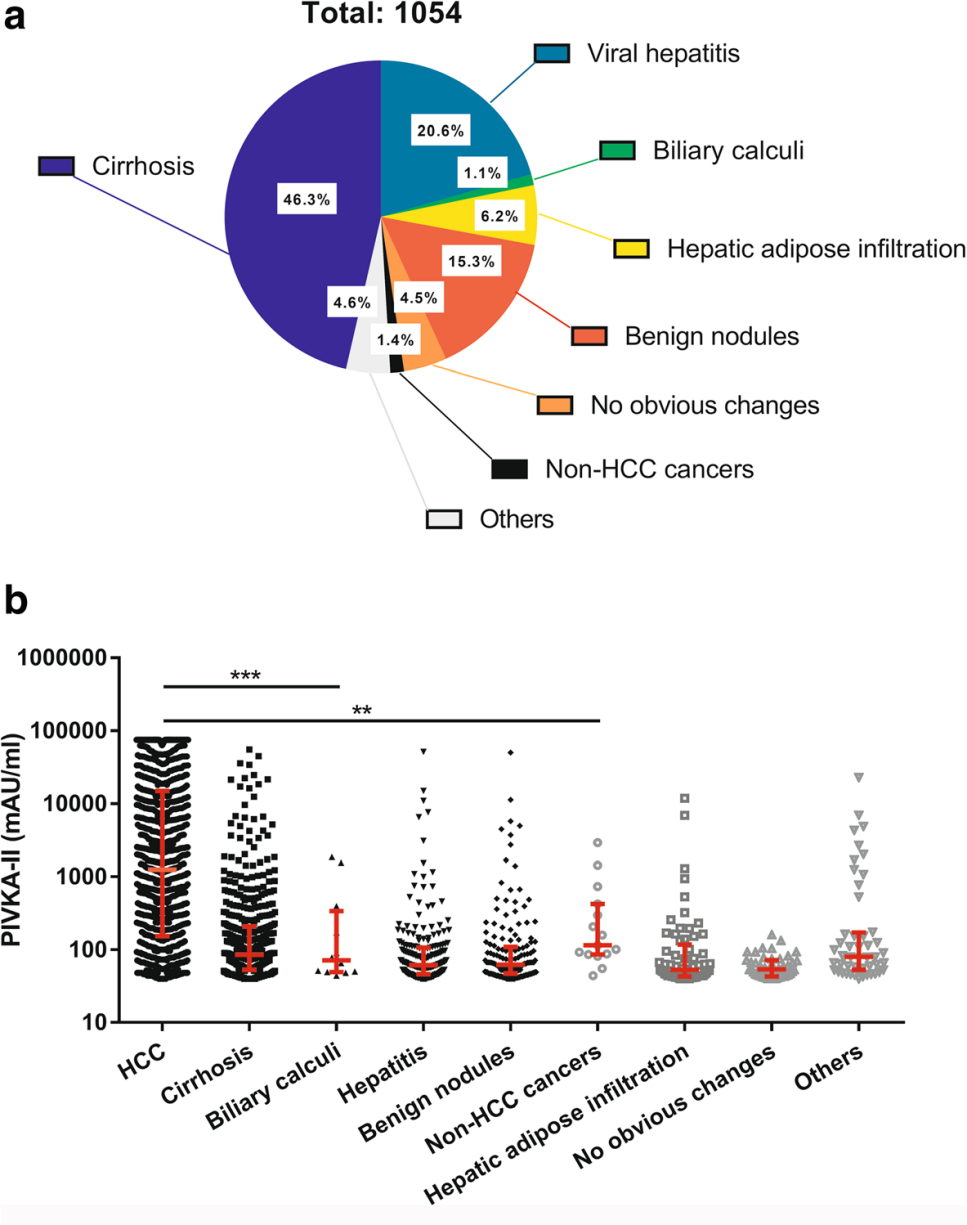
Distribution and levels of PIVKA-II in all PIVKA-II (+) enrolled patients. a Distribution of all PIVKA-II (+) enrolled patients. b Levels of PIVKA-II and their comparison among all groups. All diagnoses were concluded based on the dominant findings of image examinations or biopsy if done. Biliary calculi include calculi both in liver and gall bladder. Hepatitis includes all diseases that cause the filtration of inflammation cells or death of liver cells. Benign nodules include high-grade dysplastic nodules, low-grade dysplastic nodules, hepatic cyst, hepatic abscess, intrahepatic calcification, hepatic lipoma, liver hemangioma and other that present as benign changes of liver image. Others include pregnancy, polypi, liver transplant et al. **: <0.01, ***: <0.001 (Mann-Whitney Test)

Figure 2 shows the distribution of non-HCC cases and their levels. A total of 1054 PIVKA-II positive patients were non-HCC cases. In all these cases, cirrhosis took the largest part (46.3%), followed by hepatitis (20.6%), benign nodules (15.3%) and hepatic adipose infiltration (6.2%). Other factors that increased PIVKA-II levels included biliary calculi, non-HCC cancers. Interestingly, some PIVKA-II+ patients presented complete normal images in image examinations and this part of patients took about 4.5%. The median levels of PIVKA-II in all types of diseases were 1245.0 (interquartile range, IQR: 153.8–14,917.0), 85.0 (53.0–207.5), 71.5 (49.3–338.5), 61.0 (46.0–107.0), 62.0 (47.0–109.5), 115.0 (86.0–422.0), 53.0 (43.0–117.0), 53.5 (43.0–71.8), 80.0 (53.0–171.3) mAU/ml, respectively. Although levels of PIVKA-II elevated in other diseases, they were significantly higher in HCC group than any other groups (Mann-Whitney *P* < 0.001). However, there was no significant difference among other groups (Fig. 2b). The influence of different etiology on the level of PIVKA-II was also considered. There were 905 HBV-based HCC cases (89.1%, median PIVKA-II level: 1258.0, 156.0–14,806.0) and 65 HCV-based HCC cases (6.4%, median PIVKA-II level: 155.0, 79.5–22,773.0) and 46 other HCC cases (4.5%, median PIVKA-II level: 1261.0, 65.0–16,615.0), but there were no significant differences (Kruskal-Wallis *P* = 0.711). Among all cirrhosis cases, 396 were HBV-based (81.0%, median PIVKA-II level: 86.0, 47.5–173.8) and 56 were HCV-based (11.5%, median PIVKA-II level: 89.0, 54.0–228.0) and 37 were cirrhotic cases of other reasons (7.5%, median PIVKA-II level: 60.5, 48.3–137.5), but there were still no significant differences (Kruskal-Wallis *P* = 0.061).

**Fig. 3:**
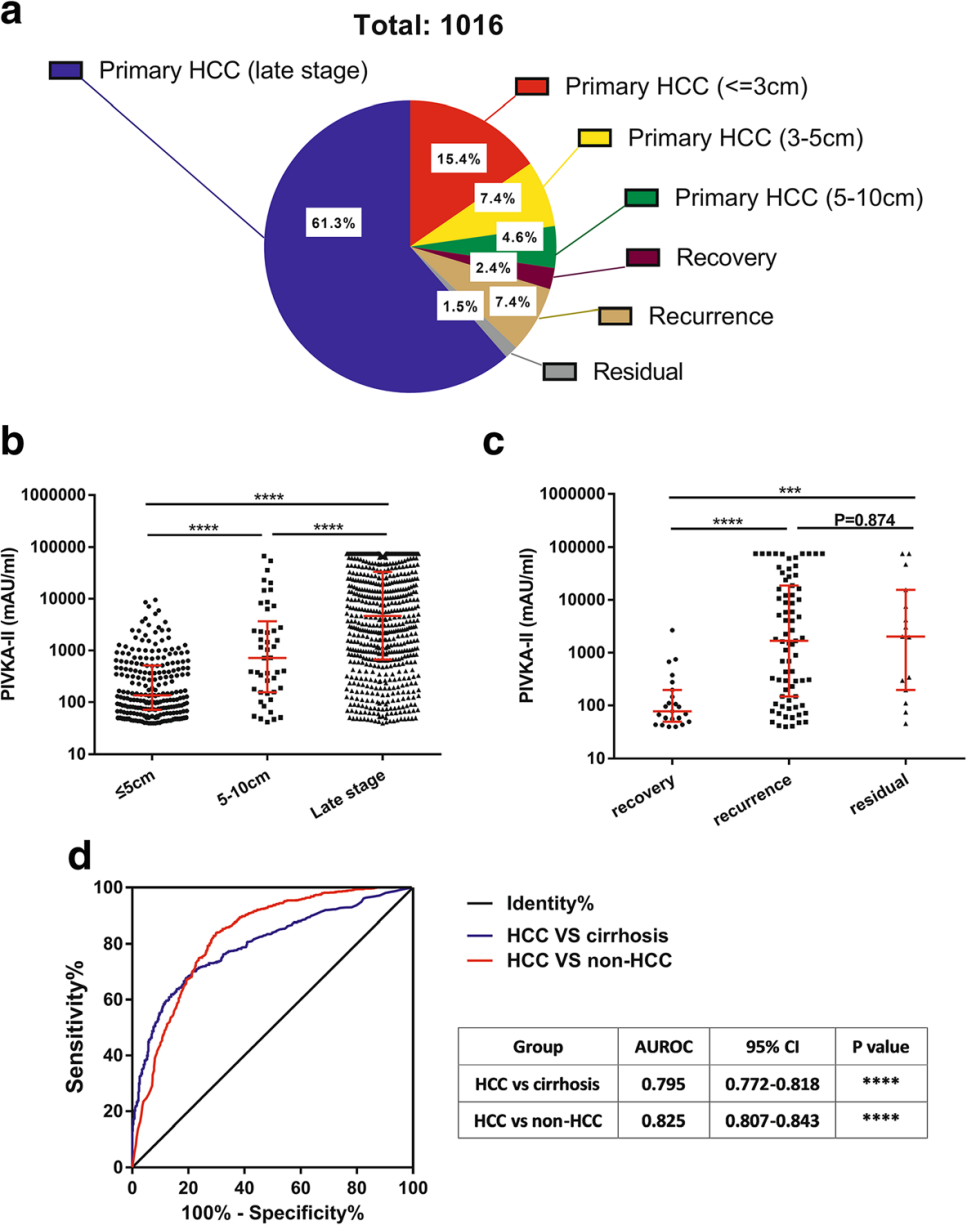
Distribution, levels and diagnostic value of PIVKA-II in HCC patients. a Distribution of all PIVKA-II (+) HCC patients. b, c Levels of PIVKA-II and their comparisons among different stages of HCC. d ROC curve for PIVKA-II in differentiating HCC from cirrhosis and non-HCC patients. ***: <0.001, ****: <0.0001 (Mann-Whitney Test)

Figure 3a shows the distribution of all cases diagnosed as HCC. In all these cases, 88.7% were primarily diagnosed and patients with advanced HCC covered 61.3% of all cases. Figure 3b and c show the mean comparison among different groups**.** Levels of PIVKA-II were significantly higher in advanced group (4650.0 mAU/ml, 667.0–33,438.0 mAU/ml) than early-stage group (tumor size < 5 cm; 104.5 mAU/ml, 61.0–348.8 mAU/ml; Mann-Whitney *P* < 0.001). The ROC curve was drawn to illustrate the effectiveness of PIVKA-II in HCC diagnosis, as shown in Fig. 3d. AUROC for HCC group and cirrhosis group was 0.795 (0.772–0.818, *P* < 0.001) and the cut-off value was 291.5 mAU/ml. AUROC for HCC group and the non-HCC group was 0.825 (0.807–0.843, *P* < 0.001) and the cut-off value for this was 303.0 mAU/ml. The other 11.3% cases were postoperative patients visiting hospital routinely and levels of PIVKA-II in recovery, recurrence and residual groups were 77.0 mAU/ml (50.0–196.0 mAU/ml), 1672.0 mAU/ml (148.0–18,683.0 mAU/ml) and 2016.0 mAU/ml (196.0–15,482.0 mAU/ml), respectively. Levels of PIVKA-II elevated significantly in recurrence and residual group than recovery group (Mann-Whitney *P* < 0.001), but there was no significant difference between recurrence group and residual group (Mann-Whitney *P* = 0.874).

### Comparison of PIVKA-II and AFP in HCC diagnosis

**Fig. 4:**
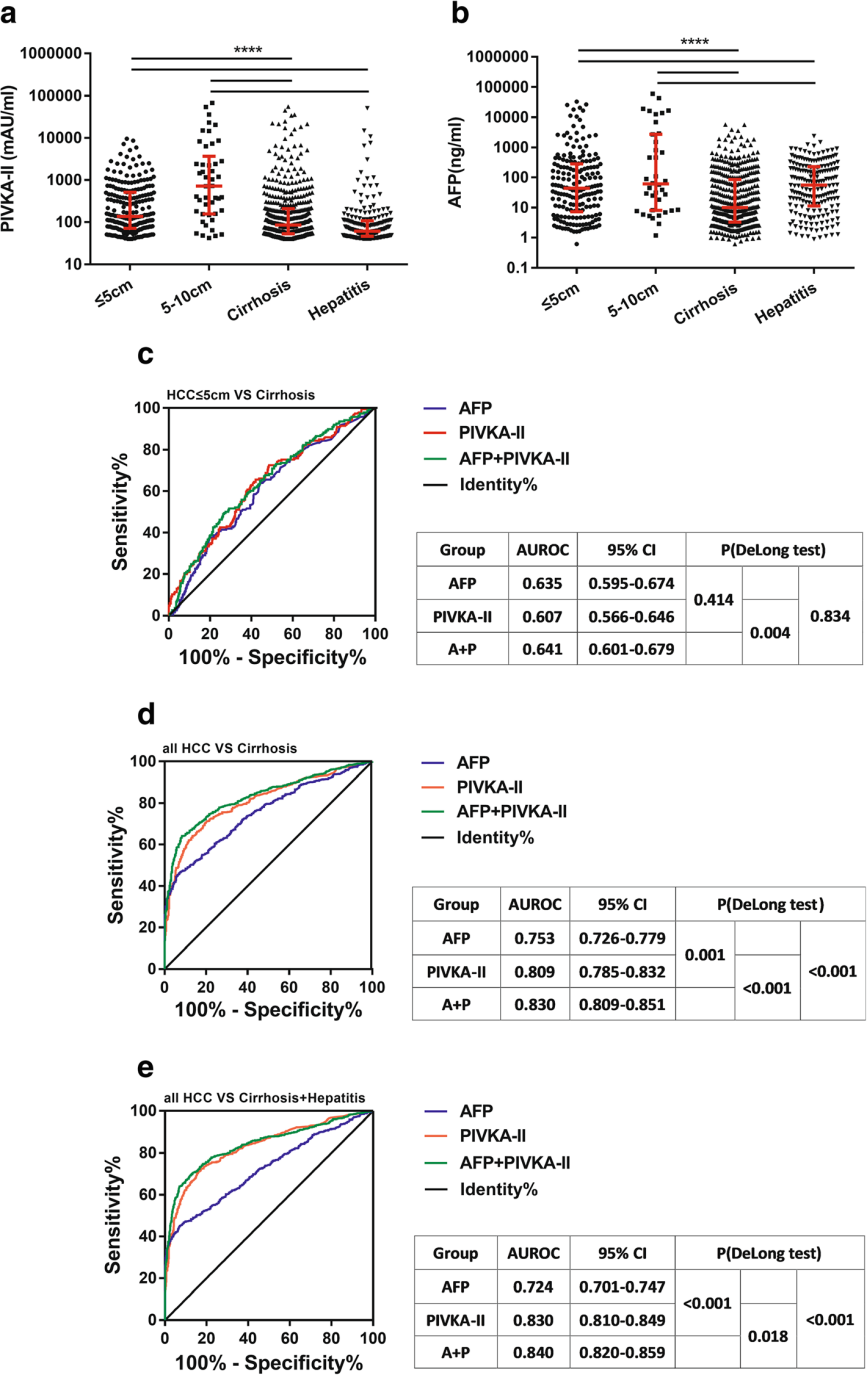
Diagnostic value of PIVKA-II in differentiating early-stage HCC from cirrhosis and hepatitis. a, b Levels of AFP and PIVKA-II in patients with early-stage HCC, cirrhosis and hepatitis. c, d, e ROC curve for PIVKA-II in differentiating early-stage HCC from cirrhosis and non-HCC patients. A + P: logAFP + 4.6*logPIVKA-II. ****: <0.0001

Figure 4a and b show the levels of PIVKA-II and AFP and their comparisons among four groups, HCC group (≤5 cm)/HCC group (5-10 cm)/cirrhosis group/hepatitis group. Both PIVKA-II and AFP levels were significantly elevated in HCC cases than cirrhosis and hepatitis groups (*P* < 0.001). Remarkably, this difference was also significant between HCC (≤5 cm) group (136.0 mAU/ml, 71.0–515.0 mAU/ml) and cirrhosis group (85.0 mAU/ml, 53–207.5 mAU/ml, *P* < 0.001). Figure 4c–e showed the ROC curve and gave a clear contrast between AFP and PIVKA-II in different groups. The combination of the two biomarkers was also evaluated. Here, we used the variable (logAFP + 4.6*logPIVKA-II) to represent the combination of AFP and PIVKA-II, as proposed by Jorge A. Marrero et al. [18]. Generally, PIVKA-II performed a better diagnostic effectiveness than AFP in differentiating HCC from non-HCC hepatic diseases and the AUROC for PIVKA-II could reach 0.8, which is obviously better than AFP (DeLong *P* = 0.001 and *P* < 0.001, respectively). In addition, the combination of the two markers could significantly improve the diagnostic performance of HCC. The AUROC for the combination was 0.830 in differentiating HCC from cirrhosis, significantly higher than AFP alone (DeLong *P* < 0.001) and PIVKA-II alone (DeLong *P* < 0.001). The AUROC for the combination was 0.840 in differentiating HCC from cirrhosis and hepatitis, significantly higher than AFP alone (DeLong *P* < 0.001) and PIVKA-II alone (DeLong *P* = 0.018). However, it seemed that both AFP and PIVKA-II could hardly differentiate early-stage HCC from cirrhosis, though the AUROC for AFP (0.635, 0.595–0.674) was slightly better than PIVKA-II (0.607, 0.566–0.646). But the difference was not significant (DeLong *P* = 0.414).

### Cumulative incidence of HCC by PIVKA-II

Levels of PIVKA-II of all CHB patients were tested in two and a half years’ time, and among them, 252 patients with more than 1 years and 3 times of follow-ups were enrolled for analysis. Based on the outcome, they were divided into HCC group and non-HCC group. Table 1 shows the baseline characteristics and Cox survival analysis of all enrolled patients. Among all the most at-risk follow-up patients, 86 cases developed into HCC during the 2 years’ follow-up. Male, age per year, ALT < 40 IU/L, TBA < 10 μmol/L, APRI < 0.5, HBV-DNA < 5*10^2^ IU/ml, HBsAg negative, HBeAg negative, PIVKA-II < 200 mAU/ml, AFP < 20 ng/ml were selected as a reference. In univariate analysis of this study, female/low level of TBA/low level of PIVKA-II/median level of AFP were protective factors. After adjustment, TBA and PIVKA-II were two variables that significantly influence the incidence of HCC for the most at-risk population and the hazard ratios were 1.918 (95% CI: 1.111–3.310, *P* = 0.019) and 0.433(95% CI: 0.277–0.678, *P* < 0.001). This was consistent with our previous study that constant high level of TBA increased the risk of HCC [25].

**Table 1:** Baseline characteristics of enrolled patients and Cox survival analysis for risk of HCC

Variables	Value^a^	Univariate analysis		Multivariate analysis	
		HR (95% CI)	*P* value	HR (95% CI)	*P* value
Gender, female	47 (18.7%)	0.524(0.271–1.014)	0.055	0.566(0.290–1.105)	0.095
Age(years)	47.4 (45.9–48.9)	1.009(0.991–1.028)	0.337		
ALT (IU/L), <40	114				
40–160	82	1.534(0.773–3.047)	0.221		
> = 160	46	1.234(0.597–2.550)	0.571		
TBA(μmol/L), <10	84				
10–100	91	2.267(1.324–3.882)	0.003	1.918(1.111–3.310)	0.019
> = 100	68	2.218(1.231–3.997)	0.008	1.654(0.897–3.053)	0.107
APRI, <0.5	40				
0.5–1.5	79	0.815(0.424–1.568)	0.541		
> = 1.5	110	1.304(0.825–2.061)	0.256		
DNA(IU/ml), <5^a^10^2^	136				
5^a^10^2^–10^6^	44	1.421(0.608–3.320)	0.417		
> = 10^6^	23	1.819(0.742–4.459)	0.191		
HBsAg, positive	198	0.529(0.244–1.147)	0.107		
HBeAg, positive	46	1.425(0.654–3.103)	0.373		
PIVKA-II(mAU/ml), <200	169	0.402(0.260–0.621)	0.000	0.433(0.277–0.678)	0.000
AFP(ng/ml), <20	115				
20–200	72	0.641(0.379–1.087)	0.099	0.611(0.357–1.045)	0.072
> = 200	42	0.700(0.391–1.251)	0.228	0.754(0.411–1.383)	0.362

*ALT* alanine aminotransferase, *TBA* total bile acid, *APRI* aspartate aminotransferase to platelet ratio index, *HBsAg* hepatitis B surface antigen, *HBeAg* hepatitis B e antigen, *PIVKA-II*, protein induced by vitamin K absence-II, *AFP* alpha-fetoprotein

^a^Some values were missing

**Fig. 5:**
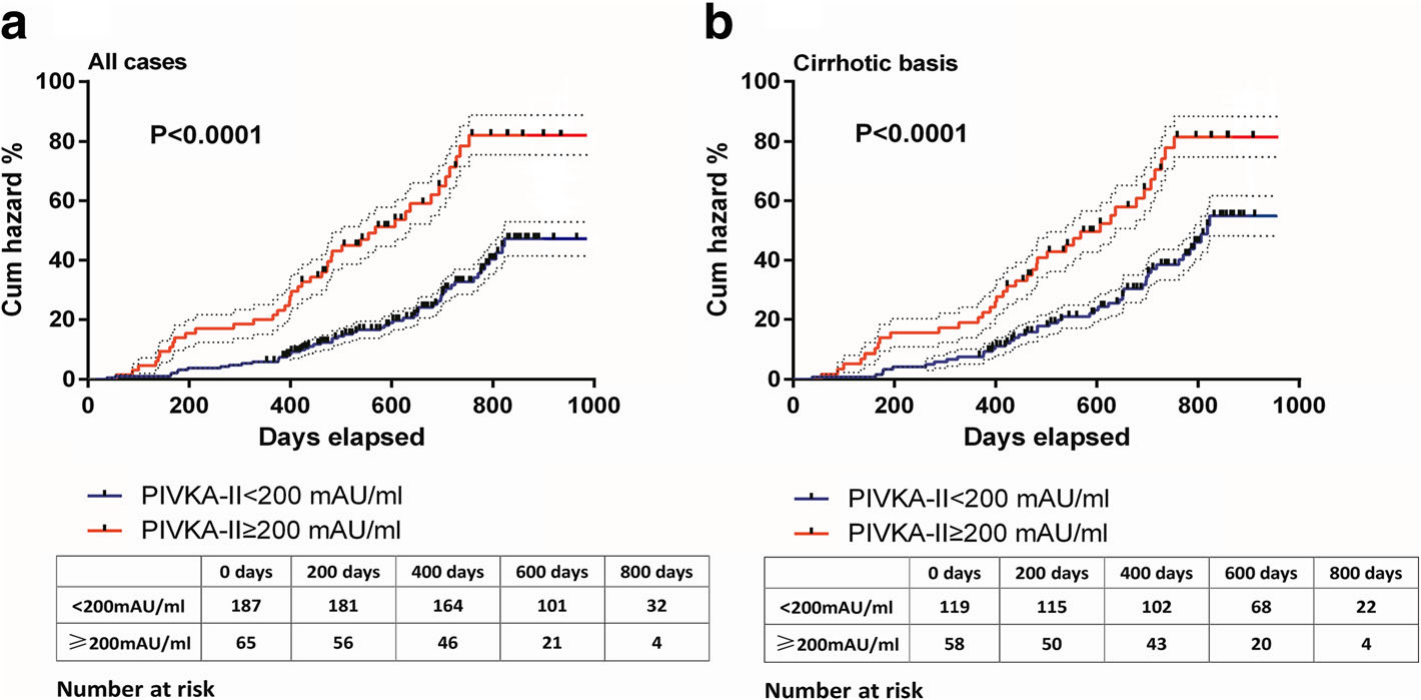
Cumulative incidence of HCC in at-risk participants. Participants were divided into two groups based on baseline PIVKA-II levels. Cumulative incidences were calculated in all enrolled groups (a) and patients with cirrhosis (b). Dashed lines are interquartile ranges

Figure 5 shows the Kaplan-Meier curve for the cumulative incidence of HCC. At-risk patients were divided into two groups: low-level group (baseline PIVKA-II < 200 mAU/ml) and high-level group (baseline PIVKA-II ≥ 200 mAU/ml), and cumulative incidence were analyzed in all at-risk patients and a sub-cohort group of cirrhotic patients. Figure 5a suggested that in all at-risk patients, the cumulative incidence was 82.0% for the low-level group and reduced significantly to 46.2% for the high-level group (log-rank *P* < 0.001) at the end of follow-up. Likewise, the cumulative incidence was 82.0% for the low-level group and reduced significantly to 54.1% for the high-level group (log-rank *P* < 0.001) at the end of follow-up, as shown in Fig. 5b.

## Discussion

The main associations for the study of liver in the world simultaneously suggest that tumor biomarkers should not be regarded as a diagnostic criterion but strongly calls for biomarkers in HCC surveillance. In the lately released American Association for the Study of Liver Diseases (AASLD) guidelines for the treatment of HCC, US with or without AFP every 6 months is the recommended strategy for HCC surveillance [26]. It should be noticed that in this guideline biomarkers are conditionally recommended for the first time, though the quality of evidence is low. European Association for the Study of the Liver (EASL) still suggests US every 6 months for HCC surveillance but emphasizes on developing accurate tumor biomarkers [27]. Asian-Pacific Association for the Study of the Liver (APASL) and JSH explicitly recommends US with tumor biomarkers as an efficient strategy for HCC [28]. Therefore, biomarkers are still critical in helping HCC surveillance and diagnosis.

Real-world researches often enrolled an abundant number of participants and a relatively less limited and strict inclusion criterion provide an actual and satisfactory external validity and could be easily spread for widely application [29, 30]. Our study for the first time analyzed the efficacy of PIVKA-II in the detection of HCC based on real-world clinical data. We hope to provide some clinical evidence for the use of PIVKA-II.

Between 2014 and 2016, 1016 patients with HCC were revealed by PIVKA-II in our hospital and among them, 230 cases would be neglected if using AFP alone. These results showed that PIVKA-II is a necessary complement to AFP and image examination in HCC surveillance. A total of 241 cases were detected in advance compared with image examination. Importantly, levels of PIVKA-II elevated over 1 year before image discovery in HCC patients. Previously, HALT-C trial and our nested case-control study evaluated the level of PIVKA-II ahead of HCC diagnosis. Both clinical research and real-world data gave the same results, indicating a proper internal and external validity of PIVKA-II. Besides, 231 patients of HCC benefited from PIVKA-II surveillance for early detection (tumor size < 5 cm) at the time when surgical interventions like hepatectomy and radiofrequency ablation were effective and even curative.

It has been suggested that levels of PIVKA-II would rise with the progression of HCC and our results gave the same conclusion [31]. But interestingly, levels of PIVKA-II in recurrence group and the residual group were significantly higher than recovery group and there was no difference between recurrence group and residual group. This phenomenon suggested that PIVKA-II could help to predict prognosis of HCC after surgery and our study gave a cut-off value of 282.5 mAU/ml. Some clinical researches have proven that PIVKA-II is a predictive factor of HCC prognosis after ablation or resection [32, 33]. Some researches go even further. *Atsushi Hiraoka* et al. used the number of tumor markers (including PIVKA-II) to predict the response to TACE [34]. *Seok-Hwan Kim* et al. found that PIVKA-II could be used for expansion of selection criteria of liver transplantation for HCC [35]. However, further large-sample multicentered studies are needed to evaluate its prognostic value and determine the cut-off.

Among all the factors that increased the levels of PIVKA-II, cirrhosis and hepatitis are familiar to us. As a result, cirrhosis and hepatitis groups are regarded as the controlled group in many studies. But as a matter of fact, any factors that damage liver cells or trigger liver cell regeneration may increase the serous level of PIVKA-II, although the mechanisms are still unclear [36]. However, clinical researches seldom pay attention to other hepatic diseases that increase levels of PIVKA-II. In our analysis, there was a large part of patients of hepatic adipose infiltration, liver cyst, liver abscess, pregnancy and so on that have elevated levels of PIVKA-II. However, compared with other groups, levels of PIVKA-II were significantly higher in HCC group, suggesting that PIVKA-II is still a biomarker proper for HCC. In addition, a high level of PIVKA-II also warns these participants of the risk of vitamin K deficiency, especially for those who were normal in image examinations. In clinical practice, further examinations may be necessary for this group of people.

Cirrhosis, HBV/HCV infection, aflatoxin B1, alcohol assumption are proven risk factors for HCC and HBeAg seropositive, high viral load, and genotype C are independent predictors of the development of HBV-related HCC. In our analysis, we provide another evidence for PIVKA-II in predicting HCC tumorigenesis. In 1 years’ time, many enrolled patients developed into HCC, because all these enrolled participants were the most at-risk population of HCC. But separately, high-level group (PIVKA-II level > 200 mAU/ml) took more risk than low-level group (*P* < 0.001) with about 80% of patients developing into HCC. This clue strongly indicated that even if PIVKA-II was not a diagnostic marker, but a high-level of PIVKA-II was still an indicator for HCC. However, although a great number of participants were enrolled in our research, the observation time was short. Further research should extend observation time and get more detailed information.

## Conclusions

This study was the first real-world research on the effectiveness of PIVKA-II in the detection of HCC. By detecting PIVKA-II, 230 AFP(−) and 241 US(−) patients were diagnosed as HCC in advance in 2 years’ time. Levels of PIVKA-II elevated more than 1 year before image diagnosis. High levels of PIVKA-II in at-risk populations were a potent indicator of developing into HCC in 2 years. Our real-world data suggested that the use of PIVKA-II improved the detection rate of PIVKA-II and was a proper complement to AFP and US.

## Abbreviations

AASLD: American Association for the Study of Liver Diseases
AFP: Alpha-fetoprotein
ALT: Alanine aminotransferase
APASL: Asian-Pacific Association for the Study of the Liver
APRI: Aspartate aminotransferase-to-Platelet Ratio Index
AUROC: Area under ROC
CHB: Chronic hepatitis B
CLIEIA: Chemiluminescence enzyme immunoassay
CMIA: Chemiluminescent microparticle immunoassay
CT: Computed tomography
DCP: Des-γ -carboxy-prothrombin
EASL: European Association for the Study of the Liver
HBeAg: Hepatitis B e antigen
HBsAg: Hepatitis B surface antigen
HBV: Hepatitis B virus
HCC: Hepatocellular carcinoma
IQR: Interquartile range
JSH: Japan Society of Hepatology
MRI: Magnetic resonance imaging
PIVKA-II: Protein Induced by Vitamin K Absence or Antagonist-II
ROC: Receiver Operating Characteristics
TBA: Total bile acid
USG: Ultrasonography
